# Analysis of the Toxicological Profile of *Heracleum sosnowskyi* Manden. Metabolites Using *In Silico* Methods

**DOI:** 10.3390/plants14213253

**Published:** 2025-10-24

**Authors:** Anna E. Rassabina, Maxim V. Fedorov

**Affiliations:** Institute for Information Transmission Problems of the Russian Academy of Sciences (Kharkevich Institute), Bolshoy Karetny per. 19, Moscow 127051, Russia

**Keywords:** chemoinformatics, furanocoumarins, *Heracleum sosnowskyi* Manden., metabolites, toxicity

## Abstract

The invasive plant *Heracleum sosnowskyi* Manden. is a valuable source of a number of bioactive metabolites that can be used in the pharmaceutical industry and medicine and may have some other applications as well. Today, there is a need to summarize data on these substances as well as analyze the toxicological profile of the metabolites of *H. sosnowskyi*. In this study, we collected a dataset of 225 metabolites of *H. sosnowskyi* from different literature sources and performed cluster analysis of their chemical structures; we revealed five main clusters of compounds: terpenoids, aromatic compounds, polyaromatic compounds, fatty acids, and furanocoumarins. In order to fill the gaps in the experimental data on the toxicity of the studied substances, we used machine learning (ML) algorithms previously designed for high-accuracy prediction of toxicity end-points. The ML-based approach allowed us to fill in up to 90% of the missing median lethal dose $LD_{50}$ (mouse) data for the studied molecules. The validity of each predicted value was confirmed by analyzing the applicability domain of the used ML models. For the calculations and ML modeling, we used the *Syntelly* chemoinformatics platform. For the most toxic compounds—hydroxycoumarins and furanocoumarins of *H. sosnowskyi*—the values for hepatotoxicity, drug-induced liver injury (DILI), cardiotoxicity, and carcinogenicity were predicted. Based on the analysis of $LD_{50}$ values for the mouse animal model, the greatest toxicity for furanocoumarins is expected with the intravenous route of administration (62–450 mg/kg), which can cause drug-induced liver injury. At the same time, the data do not show high cardiotoxicity risks for the studied furanocoumarins. Based on the presented results, we discuss prospects of using some of the compounds as pharmaceutical agents.

## 1. Introduction

Today, special attention is being paid to invasive plants that actively reproduce, disperse, and integrate into natural plant communities outside their natural range, causing significant environmental and economic damage to native species and ecosystems [1,2]. Among the numerous invasive species, there is *Heracleum sosnowskyi* Manden. (*Apiaceae*) (Sosnowski’s hogweed) (*H. sosnowskyi*) [3]. It is known that *H. sosnowskyi* has spread widely throughout several countries in Eastern and Northern Europe, as well as in the Baltic States, after it was introduced as a forage plant [4,5,6,7].

Due to their diverse chemical composition, invasive plants are able to easily adapt to various environmental conditions and successfully compete with native species. It is known that secondary metabolites of plants are important sources for the discovery of new medicines [8]. Secondary metabolites are usually multifunctional compounds capable of exhibiting various pharmacological properties. Along with many members of the *Apiaceae* family, *H. sosnowskyi* contains metabolites that exhibit photosensitizing, antioxidant, cytotoxic, antimicrobial, and antitumor activities [9]. Carbohydrates, proteins, aldehydes, esters, and phenolic compounds have been shown to occur in *H. sosnowskyi* [10,11,12]. Indeed, pectin substances and proteins were found in aqueous extracts of *H. sosnowskyi,* while alkaloids, carotene, and furanocoumarins were found in alcoholic extracts [13]. For example, 38 fatty acids have been identified in extracts obtained from various parts of *H. sosnowskyi* biomass [14]. In addition to hemicellulose polysaccharides [15], the biomass of *H. sosnowskyi* also contains glutamine, vitamins C and P, folic acid, 17 amino acids, as well as various macro- and microelements [16]. The leaves, stems, seeds, and inflorescences of *H. sosnowskyi* are of the greatest medicinal value, as they serve as sources of the most biologically active compounds [17]. This species can be considered a promising source of natural bioactive substances [18,19].

The most discussed compounds extracted from *H. sosnowskyi* are furanocoumarins, which have pronounced photosensitizing activity [20,21]. A significant diversity of furanocoumarins characterizes the phytochemical profile of *H. sosnowskyi* extract, among which are bergapten, angelicin, psoralen, xanthotoxin, pimpinellin, isopimpinellin, and sphondin [22]. The quantitative content of some furanocoumarins in a plant ranges from 0.15 mg/g to 3.14 mg/g of plant material [18]. When exposed to ultraviolet (UV) radiation, the activity of furanocoumarins increases, and, when they come into contact with human skin, they can cause severe burns [19,23].

There are linear furanocoumarins, which are derivatives of psoralen, and angular derivatives of angelicin [24,25]. It should be noted that, according to the literature, up to 400 different furanocoumarins with biological activity can be found in plants of the *Apiaceae* family alone [26].

Many metabolites of *H. sosnowskyi* are toxic to humans and animals [27]. Various toxicity end-points of some of the compounds have been studied by various animal models *in vitro* [19,28]. The toxicological properties of *H. sosnowskyi* metabolites are mainly associated with chemical classes such as alkaloids, triterpene saponins, flavonoids, and furanocoumarins [22].

Overall, there are a number of reports showing numerous toxic effects of the juice and different parts of the plant on humans and farm animals [17,29]. However, data on the toxicity end-points of the metabolites of *H. sosnowskyi* appear scattered. Therefore, it is necessary to summarize and analyze the available experimental data within the framework of an integrated approach and fill in the missing information.

There are a wide range of experimental methods for assessing toxicity, including *in vitro* and *in vivo*, but they require a large amount of time and financial resources. Therefore, due to the development of machine learning (ML) [30], the approach of predicting various toxicity parameters using *in silico* methods is currently becoming increasingly popular. Computational toxicology based on ML for predicting the toxicity of metabolites helps fill gaps in experimental data and perform an express assessment of toxicity for new compounds [31]. Chemoinformatics and ML methods make it possible to analyze large amounts of data as well as predict properties even for poorly studied compounds [32]. The quantitative structure–activity relationship (QSAR) model is widely used to predict the toxicity of compounds based on their structure [23,33]. Thus, the modular artificial intelligence platform *Syntelly* [34] allows one to analyze the physico-chemical properties as well as biological and toxicological activity of many chemical compounds [35,36].

The purpose of our study is to summarize data, cluster molecular structures, and predict the median lethal dose $LD_{50}$ (mouse) of metabolites of *H. sosnowskyi in silico*, as well as to establish hepatotoxicity, drug-induced liver injury (DILI), cardiotoxicity, and carcinogenicity of furanocoumarins from *H. sosnowskyi*. Based on these data, we discuss the risks associated with these compounds. In addition, we estimate the cost of synthesis of several selected compounds to assess their perspectives on the market.

## 2. Materials and Methods

### 2.1. Data Preparation

Data on the presence of these compounds in *Heracleum sosnowskyi* Manden. were obtained by analyzing publications contained in the PubMed, PubChem, and SciFinder databases and peer-reviewed articles with the keywords “furanocoumarins of *Heracleum sosnowskyi*”, “chemical composition of *Heracleum sosnowskyi*”, etc. The presence of compounds was previously experimentally confirmed and taken into account by us, regardless of their established amount in raw plant materials of *H. sosnowskyi*.

### 2.2. Establishing Canonical SMILES

For each substance, a search was performed for the CAS number and Canonical SMILES in the PubChem database using the Python 3.12 RDKit package. In the absence of a corresponding CAS number and canonical SMILES for the compounds [37], a structure search was performed on the Russian modular artificial intelligence platform *Syntelly* [34].

### 2.3. Clusterization of Data

Clustering was performed using the SynMap module of the *Syntelly* platform. The method of studying chemical space is based on the parametric multiscale t-SNE method in combination with differential fingerprints [38].

### 2.4. In Silico Toxicity Analysis

Aggregation and prediction of the median lethal dose $LD_{50}$ (mouse) (mg/kg) were performed on the *Syntelly* platform for mouse test objects for various routes of administration: oral, intravenous, intraperitoneal, mouse skin, subcutaneous, and intramuscular. Experimental data were collected from TOXRIC (https://toxric.bioinforai.tech, accessed on 1 July 2025), ChemIDplus, NIH (https://www.nih.gov, accessed on 1 July 2025), NCATS-Flux, PyTDC, and PubMed platforms. Analysis was performed of hepatotoxicity, drug-induced liver injury (DILI), cardiotoxicity, and carcinogenicity on the *Syntelly* platform in the Datasets module using models. CatBoost and XGBoost algorithms were used [36]. The performance of the fingerprint-based CatBoost and fragment-based XGBoost algorithms was evaluated using RMSE metric for regression tasks and ROC-AUC for classification tasks [36].

### 2.5. Assessment of the Applicability Domain

For each predicted toxicity value, the applicability domain (AD) was evaluated. AD was determined based on the structural similarity between the test molecules and the training set [39]. The molecules were projected into a 2D chemical space (SynMap module), after which the average distance to the nearest neighbors was calculated using the kNN (k = 5) method. The obtained value was normalized relative to a predefined threshold distance for each model and transformed using a Gaussian function. The final indicator of AD (0–1) reflects the reliability of the prediction: values ≥ 0.5 indicate the reliability of the prediction, values < 0.5—beyond the limits of AD. Then they are represented as percentages (*Syntelly*), where 0–20% is low reliability of prediction, 20–50% is average reliability of prediction, and 50–100% is high reliability of prediction.

### 2.6. Cost of Synthesis

The cost of synthesis was estimated by the *Syntelly* platform using the Synthesis cost module. The search was carried out using SMILES of the corresponding compound based on 1 g, and the number of stages per synthesis ranged from 1 to 6 [34].

### 2.7. Statistical Analysis

The JASP version 0.19.3 program was used for statistical data analysis. The normal distribution was analyzed using the graphical method and the Shapiro–Wilk criterion. The median toxicity was calculated as well as the 95% confidence interval for the median. The results in Supplementary Table S6 are presented as average values with standard deviations (±SDs) obtained after a five-fold check (*n* = 5).

## 3. Results

### 3.1. Dataset on Heracleum sosnowskyi Metabolites

Data on *Heracleum sosnowskyi* Manden. metabolites were collected from literature sources and peer-reviewed articles for the period 2003–2025 from the PubChem, SciFinder, and PubMed databases. The dataset includes compounds (as well as their known isomers) whose presence in *H. sosnowskyi* has been experimentally confirmed based on the literature. We compiled a dataset of 225 metabolites identified to date in *H. sosnowskyi*. According to the literature, compounds such as pabulenol, columbianetin, heraclenin, alloisoimperatorin, and heraclesol have been found in *H. sosnowskyi* in various parts of the plant [40]. Bergamottin plays a key role in the cytotoxic activity of bergamot essential oil [21] and demonstrates protumor activity [25]. The toxicity of phellopterin, scopoletin, and umbelliferone was observed, along with the toxicity of the aqueous extract of *H. sosnowskyi* [28].

For analysis, we used compounds whose presence in the plant has been experimentally proven. Therefore, some of the compounds mentioned in the literature as potential components of *H. sosnowskyi* but without a clear indication of their actual presence in the plant were not included in the dataset to avoid confusion. Table 1 presents some major compounds found in *Heracleum sosnowskyi*, including coumarins.

### 3.2. Clustering of Metabolites

The clustering method, which is widely used to evaluate the chemical space of many molecules, was also used in our study, where clusters of metabolites were formed based on their molecular similarity. Clustering was performed by the *Syntelly* platform using the SynMap module after uploading a set of molecular data to the platform in the form of standardized SMILES (Supplementary Tables S1–S5).

**Figure 1:**
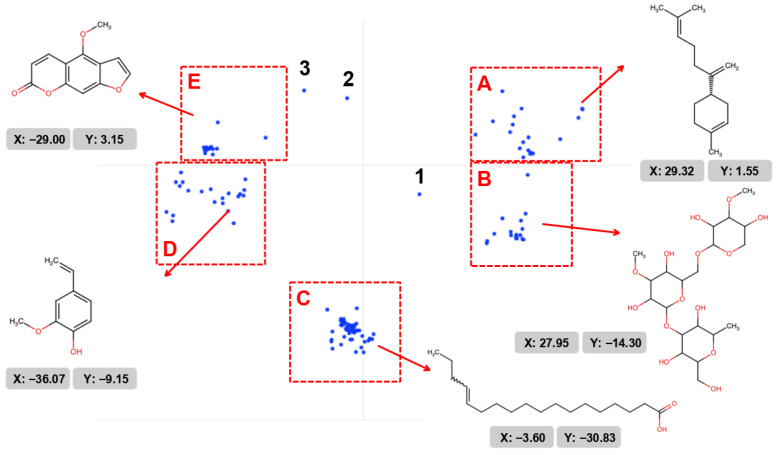
Two-dimensional clustering map of the metabolites found in *H. sosnowskyi* using the SynMap module of the *Syntelly* platform. The construction of clusters is based on the structural similarity of molecules. Clusters of molecules: A—terpenoids (isoprenoids), B—polyphenolic glycosides, flavonoids, and cyclic polyols, C—fatty acids, D—aromatic compounds, E—furanocoumarins and quinolines. The callout shows examples of connections presented in this cluster with coordinates in two-dimensional space. Connections that are not included in clusters: 1—γ-bisabolene, (z)-; 2—α-terpinolene; 3—byakangelicol.

It was found that 225 metabolites are distributed in five main clusters (Figure 1). In Cluster A, 25 compounds are mainly represented by terpenoids (isoprenoids) containing cyclohexane, bicyclic, or polycyclic fragments. Cluster B contains 22 compounds, such as polyphenolic glycosides, flavonoids, and cyclic polyols. The populous cluster C is represented by 132 fatty acids. Aromatic molecules with phenyl rings and derivatives of phenolic compounds are represented in cluster D, with a total of 21 compounds. Cluster E includes 22 compounds, mainly furanocoumarins. Cluster E also includes quininic acid and coumarin derivatives. It was found that three molecules were not included in the clusters: molecule 1 was (4Z)-1-methyl-4-(6-methylhept-5-en-2-ylidene)cyclohexene (γ-bisabolene, (z)-) a non-aromatic terpene without functional groups (–OH, –COOH) with a specific stereochemistry (Z-configuration) that was not included in cluster A. The compound 1-methyl-4-propan-2-ylidenecyclohexene (α-terpinolene) (molecule 2) is a non-aromatic terpene, which is why it did not fall into the terpenoid cluster, but, if cluster A was expanded to terpenes and their derivatives, it could well be included. And, 9-[(3,3-dimethyloxiran-2-yl)methoxy]-4-methoxyfuro[3,2-g]chromen-7-one (byakangelicol)—a furanocoumarin with an epoxy group—and the count of the epoxy group (3,3-dimethyloxiran) may differ from the furanocoumarin cluster (molecule 3) (Figure 1). We will consider the three connections that are not included in the clusters separately.

### 3.3. In Silico Toxicity Prediction and Applicability Domain Assessment

**Figure 2:**
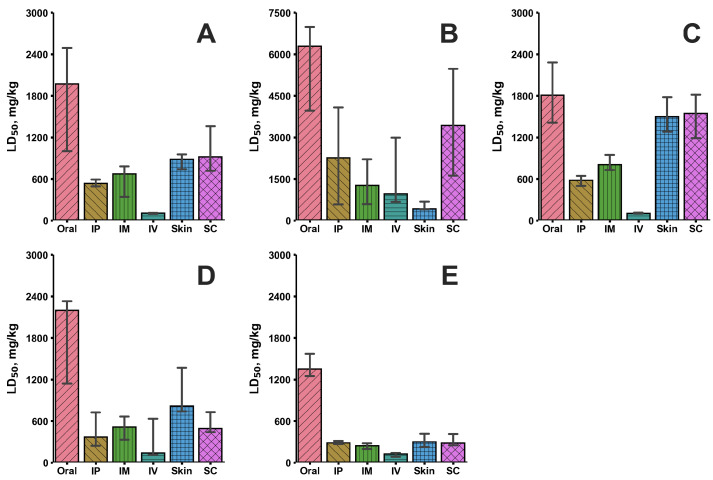
Median toxicity $LD_{50}$ (mouse) values, mg/kg for different routes of administration: Oral—oral, IP—intraperitoneal, IM—intramuscular, IV—intravenous, Skin—mouse skin, and SC—subcutaneous. Five clusters of metabolites are presented for *H. sosnowskyi*, ranking compounds by predicted toxicity, where (**A**)—terpenoids (isoprenoids), (**B**)—polyphenolic glycosides, flavonoids, and cyclic polyols, (**C**)—fatty acids, (**D**)—aromatic compounds, and (**E**)—furanocoumarins. The vertical line specifies a 95 percent confidence interval of the median.

For five clusters of compounds, the prediction of $LD_{50}$ (mouse) *in silico* was performed on test subjects in mice with various routes of administration: oral, intravenous, intraperitoneal, mouse skin, subcutaneous, and intramuscular on the *Syntelly* platform (Figure 2). In addition to the prognostic data of toxicity *in silico*, the platform aggregates experimental $LD_{50}$ (mouse) (Exp) data on some compounds.

Figure 2 shows the median toxicity values of each cluster of compounds for different routes of administration. Note that the values of the RMSE metric for regression prediction models for oral, intravenous, and intraperitoneal routes of administration averaged 45%, which indicates acceptable accuracy (Supplementary Table S6).

In a comprehensive analysis of the toxicity of compounds, we consider oral and intravenous routes of administration as the most preferred routes of drug administration. We have shown that among the five clusters of compounds in *H. sosnowskyi*, cluster E has the highest median toxicity by oral administration in mice, with $LD_{50}$ (mouse) values in the range of 423–8100 mg/kg. A cluster of fatty acids showed significant toxicity, followed by a cluster of terpenoids, followed by a cluster of aromatic compounds and polyphenolic glycosides. It is worth noting that the toxicity of the compounds depends on their route of administration, and the highest toxicity of cluster E was observed with the intravenous route of administration (62–450 mg/kg) (Figure 2). For molecules not included in the cluster, it was noted that byakangelicol has an $LD_{50}$ (mouse) of 94 mg/kg with the intravenous route of administration, for γ-bisabolene ((z)-), 401 mg/kg, and for α-terpinolene, 133 mg/kg. With the oral route of administration, these compounds did not show pronounced toxicity (over 1000 mg/kg).

For the predicted $LD_{50}$ (mouse) values, the applicability domain (AD) of the QSAR model was calculated, which defines the boundaries within which the model’s predictions are considered reliable [41] (Supplementary Table S6). If the values are in the range from 0 to 20%, then there are few molecules similar to the selected one in the training data of the model, which corresponds to low prediction reliability. The range of 20–50% corresponds to average reliability of prediction, and the range of 50–100% corresponds to high reliability. Of the five clusters of compounds in *H. sosnowskyi*, the highest AD values were observed in cluster D for aromatic compounds. It can be assumed that there are probably more aromatic compounds in the training sample of experimental data, and the model predicted the values better (Supplementary Figure S1). Cluster E comes in second place, followed by cluster B with polyphenolic compounds and flavonoids, followed by cluster A with terpenoids and cluster C with fatty acids.

### 3.4. Analysis of H. sosnowskyi Metabolite Toxicity

**Figure 3:**
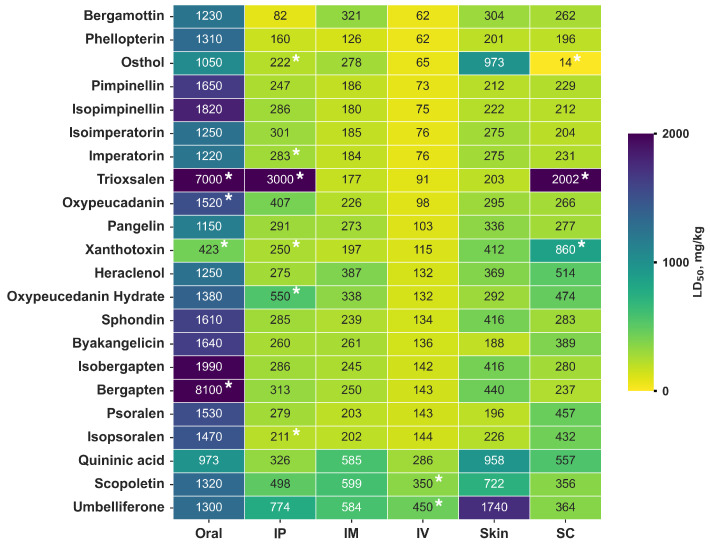
Heatmap of the toxicity spectrum $LD_{50}$ (mouse), mg/kg, of cluster E furanocoumarins found in *H. sosnowskyi*, where Oral—oral, IP—intraperitoneal, IM—intramuscular, IV—intravenous, Skin—mouse skin, and SC—subcutaneous. Both the predicted *in silico* values and the experimental values (*) are presented. The ranking is based on the intravenous (IV) route of administration. The color indicates the toxicity of the molecules: dark purple indicates low toxicity, and yellow indicates high toxicity.

For cluster E, which contains the most toxic compounds from *H. sosnowskyi*, a toxicity spectrum heatmap was constructed, where the brightest fragments correspond to the most toxic compounds (Figure 3). Recall that furanocoumarins and coumarin derivatives are the least toxic with the oral route of administration, while they exhibit the greatest toxicity with intravenous administration. The most toxic furanocoumarin is bergamottin; its intravenous $LD_{50}$ (mouse) is 62 mg/kg (AD 74%) and that of phellopterin for intravenous route of administration is 62 mg/kg (AD 60%). The least toxic are scopoletin and umbelliferone, for which the $LD_{50}$ (mouse) of an intravenous application is 350 and 450 mg/kg, respectively. When comparing the values presented by the *Syntelly* platform with the data from the articles [28], the experimental data for xanthotoxin (423 mg/kg) for the $LD_{50}$ (mouse) of furanocoumarins coincide.

For compounds of the E cluster of *H. sosnowskyi*, we were able to predict the majority of toxicity values for different routes of administration, and it is worth noting that for regression models, AD for oral, intravenous, and intraperitoneal routes of administration exceeded 50% confidence (Supplementary Figure S2). For intramuscular and mouse skin, the values of the AD did not exceed 40% on average. This was probably due to the peculiarities of the data on which the model was trained. Of all the toxicity values that were selected for complex analysis, only 10% of the total number were experimental (Figure 3), which indicates a high demand for filling in the missing data.

### 3.5. Prediction of General Toxicity In Silico

In addition to analyzing the toxicity of $LD_{50}$ (mouse), we aggregated experimental data (Exp) and obtained prognostic data *in silico* on hepatotoxicity, drug-induced liver injury (DILI), cardiotoxicity, and carcinogenicity of cluster E (Table 2). For the binary classification of toxic/nontoxic, the values of the ROC-AUC ranged from 79% to 93% (Supplementary Table S6), which shows the high efficiency of prediction models. It is shown that furanocoumarins from *H. sosnowskyi* can cause drug-induced liver injury, while they have a low risk of cardiotoxicity (Table 2). As a result, it is predicted that 5 out of 22 compounds do not have hepatotoxicity. It is worth noting that the AD of the hepatotoxicity parameter for many values was higher than 60%, with the exception of 4-[(3,3-dimethyloxiran-2-yl)methoxy]furo[3,2-g]chromen-7-one ((R)-Oxypeucedanin) and 6-methoxyquinoline-4-carboxylic acid (quininic acid), for which the AD of hepatotoxicity was 38% and 29%, respectively. We found an absence of carcinogenicity for five compounds in cluster E, which had an AD over 45%.

### 3.6. Estimation of the Cost of Synthesis

Using the Synthesis cost module of the *Syntelly* platform, the synthesis cost was estimated for three representative compounds from cluster E. The highest estimated cost for the synthesis of xanthotoxin from 2-hydroxybutanedioic acid and 2,3-dihydro-1-benzofuran-6,7-diol was USD 311 per 1 g of product. The cost of psoralen synthesis turned out to be about 12.5 times lower than that of xanthotoxin. Interestingly, cost estimations correlate with the content of these compounds in the plant material of *H. sosnowskyi*: xanthotoxin, which is more expensive to synthesize, has a higher natural content (0.8 mg/g) compared to psoralen (0.2 mg/g) [18].

The lowest estimation of the synthesis cost was found for hydroxycoumarin umbelliferone (a pathway from benzoyl chloride would cost USD 0.19 per 1 g of the product). Given its key role in the biosynthesis of coumarins and its importance in applications [42], it is of the greatest interest as a promising object for chemical synthesis, with the possibility of subsequent modification into more complex derivatives, for example, furanocoumarins. At the same time, umbelliferone does not show cardiotoxicity and carcinogenicity (Table 2), which correlates well with experimental observations of its low toxicity [43].

## 4. Discussion

Currently, there is a need for a comprehensive analysis of the toxicological profile of *H. sosnowskyi* metabolites because of (1) risks associated with the wide and uncontrolled distribution of this invasive plant, and (2) the plant may become a promising source of biologically active substances. The toxicity data on the compounds available in the literature are incomplete and fragmented. Therefore, we believe that the presented study fills some of the gaps, and it may form a deeper understanding of the chemical composition of the invasive plant.

Due to the growing popularity of using QSAR [44], predictive models should be validated, and the most important element of this process is a clear definition of the model’s applicability domain (AD) [45]. The AD is calculated based on the comparison of the predicted molecule with the molecules of the training sample for each model. Thus, the assumption here is that if the molecule is structurally similar to the training sample, then we believe that the model can make predictions for it. The molecules of the training sample and the predicted molecule are projected onto a map of the chemical space [46] (a 2D model used in the SynMap module). Then the kNN method is used—the average distance from the predicted molecule to the five nearest neighbors is calculated. For each model, the distance is defined in advance for properties, the boundary beyond which the model is considered inapplicable. The calculated average distance is normalized relative to the boundary, and then a Gaussian function is applied to this value. As a result, we obtain the values of the AD. For metabolites of a phenolic nature, toxicity values with a high AD index can be obtained. We confirmed that furanocoumarins from *H. sosnowskyi* are the most toxic molecules among the compounds of a phenolic nature. The analysis of clusters of terpenoids and fatty acids seems promising, but their detailed analysis was outside the scope of the presented work.

We collected a dataset of 225 metabolites from *H. sosnowskyi*, which contains standardized SMILES of molecules and SynID (Supplementary Tables S1–S5). The dataset contains both *in vitro* experimental data (aggregated data obtained by HPLC, GC-MS/MS) and predicted *in silico* data. However, the authors do not exclude the possibility that *H. sosnowskyi* may contain a wide range of other compounds that need to be investigated in order to form a complete picture of the toxicity of all secondary metabolites, and this dataset will be updated in the future as soon as new experimental data are found in the literature.

Our analysis revealed that phenolic compounds and lipids constituted the largest proportion of metabolites in the dataset, which can be attributed to their high abundance in *H. sosnowskyi* [14,47]. It was previously shown that leaf extracts had a high content of triterpenes (7.4 mg/g DW) (Table 1) [14], and the total furanocoumarin content in the leaves of *H. sosnowskyi* was >6.4 mg/g [18].

We reported the toxicological profile of furanocoumarins, quininic acid, and some coumarins found in *H. sosnowskyi*. At the same time, we did not take into account the synergism of compounds *in vivo*, and there are no data on chronic toxicity.

Furanocoumarins are known to be actively used as pharmaceuticals, despite the fact that they have pronounced toxicity [48,49]. To assess the potential use of raw plant materials as a source of secondary metabolites for the production of medicines, it is important to precisely identify the different types of general toxicity (hepato-, cardio-, carcinogenicity) of all furanocoumarins studied. Analysis of hepatotoxicity, cardiotoxicity, including DILI [50,51], and carcinogenicity of poorly studied furanocoumarins is necessary for assessing the risks associated with pharmaceutical preparations based on raw plant materials from *H. sosnowskyi*. The DILI parameter will allow us to evaluate the effectiveness of the use of furanocoumarins as medicines. The values of hepatotoxicity for 15 furanocoumarins were revealed (Table 2).

The calculation of the cost of hydroxycoumarin and furanocoumarin synthesis will make it possible to evaluate the possibility of obtaining compounds using chemical synthesis. We showed that the cost of these compounds varies from USD 0.19 to 311 per 1 g. The synthesis of umbelliferone is potentially cheap and can be scaled for practical applications. Thus, we showed that there is variation in the cost of synthesis of furanocoumarins and their derivatives, which must be taken into account during further extraction or synthesis.

Certainly, the data obtained on *in silico* toxicity require confirmation by *in vitro* methods, but this comprehensive study will optimize the selection of promising compounds for subsequent stages of *in vivo* testing and reduce time and financial costs by minimizing experiments. Thus, the integration of *in silico* and *in vitro* methods will provide a more complete and reliable assessment of the toxicity of metabolites, which is especially important in the development of drugs and other bioactive compounds.

## 5. Conclusions

We compiled a dataset of 225 metabolites reported in *H. sosnowskyi* to date, based on data in the literature. We provide their chemical structures and discuss their toxicological properties in detail using both available experimental data and ML-based estimations in those cases where experimental data were not available. This provides a source of data for future studies of the chemotype of the plant. The dataset also gives some background for a systematic analysis of its usage in the biotech and pharmaceutical industries. It may be used as a benchmark for future studies of the mechanisms of its profound invasive capabilities compared to other relative species.

As a result of the conducted analysis, it was established that the main toxicological hazard of *H. sosnowskyi* is associated with furanocoumarins and coumarin derivatives. The highest toxicity of the selected furanocoumarin cluster was observed for intravenous administration in $LD_{50}$ (mouse), ranging from 62 to 450 mg/kg. The data obtained are of fundamental and practical importance, important for assessing the risks of using *H. sosnowskyi* metabolites as medicines, as well as for obtaining their synthetic analogues.

## Figures and Tables

**Table 1:** Content of chemical substances in different parts of *Heracleum sosnowskyi*.

Parts of the Plant	Substance	Content, mg/g *	References
	Triterpenes	7.4	[14]
Leaves	Terpenes	0.6	[14]
	Furanocoumarins	>6.4	[18]
Seeds	Carboxylic acids	21.0	[14]
	Coumarins	22.8	[14]
	Carboxylic acids	1.1	[14]
Stems	Terpenes	<0.1	[14]
	Alcohols	<0.1	[14]

* Dry weight of raw materials (DW).

**Table 2:** Data on hepatotoxicity, drug-induced liver injury (DILI), cardiotoxicity, and carcinogenicity of cluster E compounds isolated from *Heracleum sosnowskyi* and applicability domain (AD) values in % for each predicted value.

Substance	Hepatotoxicity		DILI		Cardiotoxicity		Carcinogenicity	
	Effect	AD,%	Effect	AD,%	Effect	AD,%	Effect	AD,%
Bergamottin ^a^	Toxic	90	Toxic	41	Nontoxic	67	Nontoxic	48
Phellopterin ^a^	Toxic	91	Toxic	41	Nontoxic	PyTDC	Nontoxic	58
Osthol ^b^	Toxic	TOXRIC *	Toxic	48	Nontoxic	PyTDC	Toxic	79
Pimpinellin ^a^	Toxic	88	Toxic	40	Nontoxic	51	Nontoxic	51
Isopimpinellin ^a^	Toxic	79	Toxic	37	Nontoxic	PyTDC	Nontoxic	45
Isoimperatorin ^a^	Toxic	TOXRIC	Toxic	46	Nontoxic	PyTDC	Toxic	76
Imperatorin ^a^	Toxic	TOXRIC	Toxic	43	Nontoxic	PyTDC	Nontoxic	61
Trioxsalen ^a^	Toxic	TOXRIC	Toxic	53	Nontoxic	NCATS-Flux	Toxic	88
Oxypeucedanin ^a^	Toxic	38	Toxic	29	Nontoxic	PyTDC	Toxic	26
Pangelin ^a^	Toxic	61	Toxic	41	Nontoxic	PyTDC	Nontoxic	77
Xanthotoxin ^a^	Toxic	89	Toxic	41	Nontoxic	PyTDC	Toxic	45
Heraclenol ^a^	Nontoxic	92	Toxic	41	Nontoxic	PyTDC	Nontoxic	58
Oxypeucedanin Hydrate ^a^	Nontoxic	90	Toxic	41	Nontoxic	PyTDC	Nontoxic	57
Sphondin ^a^	Toxic	89	Toxic	46	Nontoxic	PyTDC	Toxic	61
Byakangelicin ^a^	Nontoxic	89	Toxic	40	Nontoxic	PyTDC	Nontoxic	47
Isobergapten ^a^	Toxic	82	Toxic	42	Nontoxic	54	Toxic	47
Bergapten ^a^	Toxic	TOXRIC	Toxic	41	Nontoxic	NCATS-Flux	Toxic	48
Psoralen ^a^	Toxic	TOXRIC	Toxic	51	Nontoxic	PyTDC	Toxic	79
Isopsoralen ^a^	Toxic	93	Toxic	42	Nontoxic	23	Toxic	54
Quininic acid ^c^	Nontoxic	29	Toxic	63	Nontoxic	66	Nontoxic	49
Scopoletin ^b^	Nontoxic	TOXRIC	Toxic	51	Nontoxic	PyTDC	Nontoxic	PyTDC
Umbelliferone ^b^	Toxic	TOXRIC	Toxic	57	Nontoxic	67	Nontoxic	46

^a^ Furanocoumarin; ^b^ coumarin; ^c^ cyclohexanecarboxylic acid. * TOXIC and PyTDC are special designations used in the model.

## Data Availability

Most of the data are provided in the Supplementary Materials to this paper. Other data supporting the results of this study can be obtained from the corresponding authors upon reasonable request.

## Supplementary Materials

The following supporting information can be downloaded at https://www.mdpi.com/article/10.3390/plants14213253/s1. Tables S1–S5: Standardized SMILES and corresponding identification numbers (SynID) for clusters A–D; Table S6: Main model quality parameters; Figure S1: Heatmap of the values of the applicability domain (AD, %) for each predicted acute $LD_{50}$ (mouse) for cluster D of *Heracleum sosnowskyi* with various routes of administration. EXP is the experimental values aggregated from the ChemIDplus, Toxic, PyTDC, ECHA, NIH, and PubMed platforms. The value 0 indicates the inability to predict the values for these molecules; Figure S2: Heatmap of the values of the applicability domain (AD, %) for each predicted acute $LD_{50}$ (mouse) cluster E of *Heracleum sosnowskyi* with various routes of administration. EXP is the experimental values aggregated from the ChemIDplus, Toxic, PyTDC, ECHA, NIH, and PubMed platforms. The value 0 indicates the inability to predict the values for these molecules.
