# Supplementary material for: Analysis of the Toxicological Profile of Heracleum sosnowskyi Manden. Metabolites Using In Silico Methods
Source: Plants (Basel). 2025 Oct 24;14(21):3253. doi: 10.3390/plants14213253 (PMC12610272; doi:10.3390/plants14213253)
Supplement: Supplementary file 1 [file plants-14-03253-s001.zip › plants-3875800-supplementary.pdf]

plants

MDPI

Supplementary Materials:

Table S1. Standardized SMILES and corresponding identification numbers (SynID) for cluster A

№	SMILES	SynID
1.	C=C1CCC2CC1C2(C)C	17003038
2.	CCC(CCC(C)C1CCC2C3=CCC4CC(O)CCC4(C)C3CCC21C)C(C)C	17445660
3.	CC1=CCC(O)(C(C)C)CC1	16999638
4.	CCC(C=CC(C)C1CCC2C3CC=C4CC(O)CCC4(C)C3CCC12C)C(C)C	17099622
5.	CC(C)C(C)CCC(C)C1CCC2C3CC=C4CC(O)CCC4(C)C3CCC12C	17270083
6.	C=C(C)C1CC=C(C)CC1	17009822
7.	CC1=CCC2(C(C)C)CC12	17005729
8.	CC(C)=CCC[C@@H](C)[C@H]1C=CC(C)=CC1	68392366
9.	CC(C)(O)[C@@H]1Cc2c(ccc3ccc(=O)oc23)O1	17072397
10.	C=C1CC[C@@H]2[C@H]1[C@H]1[C@H](C(C)C)CC[C@]12C	156324613
11.	C=C(CCC=C(C)C)[C@@H]1CC=C(C)CC1	7538864
12.	CC1(O)CCC(C(C)(C)O)CC1	16995382
13.	CC1=CCC2CC1C2(C)C	16995385
14.	C=C(C)[C@@H]1CC[C@@]2(C)CCC=C(C)[C@@H]2C1	68131160
15.	CC(C)[C@H](C)CC[C@@H](C)[C@H]1CC[C@H]2[C@@H]3CC=C4C[C@@H](O)CC[C@]4(C)[C@H]3CC[C@]12C	156241383
16.	CC[C@H](/C=C/[C@@H](C)[C@H]1CC[C@H]2[C@@H]3CC=C4C[C@@H](O)CC[C@]4(C)[C@H]3CC[C@]12C)C(C)C	156241406
17.	C=C1CCC2(C(C)C)CC12	17006616
18.	CC(C)=CCCC(C)C1C=CC(C)=CC1	17445557
19.	C=C1/C=C/[C@H](C(C)C)CC/C(C)=C/CC1	8776368
20.	CC[C@H](CC[C@@H](C)[C@H]1CC[C@H]2[C@@H]3CC=C4C[C@@H](O)CC[C@]4(C)[C@H]3CC[C@]12C)C(C)C	156241408
21.	CC1=CCC2(C)CCC(C(C)C)=C2CC1	17147083
22.	CC[C@H](CC[C@@H](C)[C@H]1CC[C@H]2C3=CC[C@H]4C[C@@H](O)CC[C@]4(C)[C@H]3CC[C@]12C)C(C)C	156333942
23.	CC(C)(O)[C@@H]1Cc2cc3ccc(=O)oc3cc2O1	17289929
24.	C=C1CC/C=C(\C)CC[C@@H]2[C@@H]1CC2(C)C	8746473
25.	C=Cc1c(C)c2cc3nc(c4c5[n-]c(cc6nc(cc1[n-]2)C(C)=C6CC)c(C)c5C(=O)[C@@H]4C(=O)OC)[C@@H](CCC(=O)OC/C=C(\C)CCCC(C)CCCC(C)CCCC(C)C)[C@@H]3C.[Mg+2]	81132483

Table S2. Standardized SMILES and corresponding identification numbers (SynID) for cluster B

№	SMILES	SynID
1.	OC[C@H]1OC(O)[C@H](O)[C@@H](O)[C@H]1O	16994811
2.	O=C1O[C@H]([C@@H](O)CO)C(O)=C1O	155854192
3.	C[C@@H]1O[C@@H](OC[C@H]2O[C@@H](Oc3c(-c4ccc(O)c(O)c4)oc4cc(O)cc(O)c4c3=O)[C@H](O)[C@@H](O)[C@@H]2O)[C@H](O)[C@H](O)[C@H]1O	8746003
4.	CCC1OC(OCC2OC(OCC3OC(OCC4OC(OCC5OC(OCC6OC(C)C(O)C6C)C(O)C5OC5OC(CO)C(O)C5O)C(O)C4O)C(O)C3OC3OC(CO)C(O)C3O)C(O)C2OC2OC(CO)C(O)C2O)C(O)C1O	144616507
5.	C[C@@H]1O[C@@H](O[C@@H]2[C@H](O)[C@@H](O)[C@@H](O)O[C@@H]2C(=O)[O-])[C@H](O)[C@H](O)[C@H]1O	159542560
6.	OC[C@H]1OC(O)[C@@H](O)[C@@H](O)[C@@H]1O	17006741
7.	OC1OC[C@@H](O)[C@H](O)[C@H]1O	17109915
8.	OC1C(O)C(O)C(O)C(O)C1O	16989969
9.	O=C(OC[C@H]1O[C@@H](OC(=O)c2cc(O)c(O)c(OC(=O)c3cc(O)c(O)c(O)c3)c2)[C@H](OC(=O)c2cc(O)c(O)c(OC(=O)c3cc(O)c(O)c(O)c3)c2)[C@@H](OC(=O)c2cc(O)c(O)c(OC(=O)c3cc(O)c(O)c(O)c3)c2)[C@@H]1OC(=O)c1cc(O)c(O)c(OC(=O)c2cc(O)c(O)c(O)c2)c1)c1cc(O)c(O)c(OC(=O)c2cc(O)c(O)c(O)c2)c1	53622940
10.	C=C1C=C[C@@H]([C@@H](C)CCC=C(C)C)CC1	75341385
11.	CCCCCCCCCCCCC1CCC(=O)O1	17077488
12.	CC1OC(OCC2OC(Oc3c(-c4ccc(O)c(O)c4)oc4cc(O)cc(O)c4c3=O)C(O)C(O)C2O)C(O)C(O)C1O	8755466
13.	CC[C@@H]1O[C@@H](OC[C@@H]2O[C@@H](OC[C@@H]3O[C@@H](OC[C@@H]4O[C@@H](OC[C@@H]5O[C@@H](OC[C@@H]6O[C@@H](C)C(O)[C@@H]6C)C(O)[C@@H]5O[C@@H]5O[C@@H](CO)[C@@H](O)C5O)C(O)[C@@H]4O)C(O)[C@@H]3O[C@@H]3O[C@@H](CO)[C@@H](O)C3O)C(O)[C@@H]2O[C@@H]2O[C@@H](CO)[C@@H](O)C2O)C(O)[C@@H]1O	159542358
14.	OC[C@H]1OC(O)[C@H](O)[C@@H](O)[C@@H]1O	16994595
15.	O=C(O)[C@H]1OC(O)[C@H](O)[C@@H](O)[C@H]1O	17380404
16.	OC1OC[C@H](O)[C@H](O)[C@H]1O	17380386
17.	OC1[C@@H](O)[C@H](O)O[C@H](O)[C@H]1O	160342034
18.	COC1C(O)COC(OCC2OC(OC3C(O)C(C)OC(CO)C3O)C(O)C(OC)C2O)C1O	91506637
19.	OCC1O[C@@H](O[C@@H]2C(CO)OC(O)C(O)C2O)C(O)C(O)C1O	53672256
20.	C[C@@H]1OC(O)[C@H](O)[C@H](O)[C@H]1O	17012435
21.	CC(C)(O[C@@H]1O[C@H](CO)[C@@H](O)[C@H](O)[C@H]1O)[C@H]1Oc2ccc3ccc(=O)oc3c2[C@H]1O	23759896
22.	COC1OC(CO)C(OC2OC(CO)C(OC3OC(CO)C(OC)C(O)C3O)C(O)C2O)C(O)C1O	30395376

Table S3. Standardized SMILES and corresponding identification numbers (SynID) for cluster C

№	SMILES	SynID
1.	CCCCCCCCCC=O	16996864
2.	CCCCCCOC(=O)C(C)CC	17012024
3.	CCCCCCCCO	155804781
4.	CC/C=C\C/C=C\C/C=C\CCCCCCCC(=O)O	8746076
5.	CCCCCCCC/C=C\CCCCCCCCO	8749162
6.	CCCC(=O)O	155809484
7.	CCCCCCCCC=CCCCCCCCCCC(=O)O	17116956
8.	CCCCCCC=CCCCCCCCCCC(=O)O	17099436
9.	CCCCCCC=CCCCCCCCCC(=O)O	77700629
10.	CCCCCCCCCCCCCCCCCCCCCC(=O)O	16996904
11.	CCC/C=C/CCCCCCCCCCCC(=O)O	53429491
12.	CCCCC/C=C/C/C=C\CCCCCCCC(=O)O	8747526
13.	CCCCC=CC(=O)O	17016035
14.	CCCCCCCCCOC(C)=O	16996853
15.	O=C(O)CCC(=O)O	155861956
16.	C/C(=C\CO)CCC[C@H](C)CCC[C@H](C)CCCC(C)C	8745745
17.	CCCCCCCCCCCCCCCCCCCCCCCCCCCCC	17000774
18.	C=CC(=C)CC/C=C(\C)CCC=C(C)C	8746475
19.	C=CC(=C)CCCC(C)CCCC(C)CCCC(C)C	16998920
20.	CCCCCCCC(=O)O	155804958
21.	CC(=O)CCCC(C)CCCC(C)CCCC(C)C	16998887
22.	CCCCCCC=CCCCCCCCC(=O)O	16993535
23.	CCCCCCC(C)O	155804780
24.	C=C[C@@H](O)C#CC#CC/C=C\CCCCCCC	8746209
25.	CCCCCCCCCCCCCCCCC(=O)O	16998939
26.	C/C(=C\CO)CCCC(C)CCCC(C)CCCC(C)C	8811916
27.	C/C=C(C)/C=C/C=C(C)C	8813894
28.	CCCCCCCC/C=C\CCCCCC(=O)O	8776952
29.	C=C/C(C)=C\CC=C(C)C	8778386
30.	CCCCCCCCCCCCCCCCCCCCCCCCCCO	17050608
31.	C=CC(C)(O)CCC=C(C)C	16995284
32.	CCCCCCCCCCC(=O)O	16996869
33.	CCCCCCCCCCCCCCCCCCCCCCCCCCCCCC	16999438
34.	CCCCCCCCCCCCCCCCCCCCCCCCCCCC	17000773
35.	CCCCCCCCC=CCCCCCCCCO	16997515
36.	CCCCCCCC=O	16989591
37.	CCCCCCCCCCCCCCCCCCCCCCCCCCCCCCC	17000775
38.	CCCCCCCCOC(=O)C(C)C	17043923
39.	CCCCCCCCC=CCCCCCC(=O)O	17464689
40.	C/C=C(/C)C(=O)O	17553263
41.	CCCCCCCC/C=C\CCCCCCCC(=O)O	17385424
42.	CCCCCCCCCCC/C=C\CCCCC(=O)O	8746189
43.	CCCCC/C=C\C/C=C\CCCCCCCC(=O)O	8745756
44.	CCCCCCCCCCCCCCCCCCCCCC(=O)O	17005029
45.	CCC/C=C\CCCCCCCCCC(=O)O	68132547

46.	CCCCCCCCCCCCCCCCCCCCCCCCCCCCCCCCCC	17013472
47.	CCCCCCCCCCCCCCCCCCCCCCCC(=O)O	16999607
48.	CCCCCCCCCCCCCCCCCCCCCCCCC	17000770
49.	CCCCCCCCOC(=O)C(C)CC	17444859
50.	CC(C)CC(=O)O	16998907
51.	O=C(O)CCCCCCC(=O)O	16998931
52.	CCCCCC/C=C\CCCCCCCC(=O)O	17385423
53.	CCCCCCCCOC(=O)CCCCC	17008634
54.	CCCCC/C=C\CCCCCCCCCCC(=O)O	17385895
55.	CC(C)CCCCCCCCCCCCCC(=O)O	17136243
56.	CC(=O)O	155824580
57.	CCCCCCC=CCCCCCCCCCCCC(=O)O	77695355
58.	CCCCCCCCC(=O)O	16996847
59.	C=CC(CCCCC)OC(C)=O	17005064
60.	CCCCCCCCCCC=CCCCCCCCC(=O)O	17074231
61.	CCCCCCO	155805535
62.	CCCCCCCCCCCC=CCCCCC(=O)O	17000017
63.	CCCCCC/C=C\CCCCCCCCC(=O)O	8771786
64.	CCCCCCCC/C=C\CCCCCCCCCC(=O)O	8747497
65.	CCCCCCCCCC/C=C\CCCCCCCC(=O)O	8747496
66.	CCCCCCCCC=CCCCCCCCCC(=O)O	17212139
67.	CCCCCCCCOC(=O)CC(C)C	17192216
68.	CCCCCCOC(=O)CC(C)C	17044317
69.	CCCCCCOC(C)=O	16997498
70.	O=CCCCCCCCC(=O)O	17057183
71.	CC(=CCO)CCCC(C)CCCC(C)CCCC(C)C	17119304
72.	CCCC=CCCCCCCCCCCCCC(=O)O	4119705
73.	CCCCCCCCCCC/C=C/CCCCCC(=O)O	8771865
74.	C=C/C(C)=C/CC=C(C)C	8746505
75.	C=CCCCCCCCCCCCCCCCCCCCCCCC	17452261
76.	CC/C=C/CCCCCCCCCCCCCC(=O)O	8747492
77.	C=CCCCCCCCCCCCCCCCCCCCCCCCCC	17452260
78.	CCC=CCC=CCC=CCCCCCCCC(=O)O	16989946
79.	CCCCCCCCCCCCCCCCCCCCCCCCCC(=O)O	16998943
80.	CCCCCCCCCCCCCCCCCCCCCCCCCCCCCC	17000885
81.	CCCCCCCCCCCCCCCCCCCCCCCCCCC	17000019
82.	CCCCCCCCCCCCCCCCCCCC(=O)O	16998941
83.	CCCCCC/C=C\CCCCCCCCCC(=O)O	8747490
84.	CCCCCCCCOC(=O)CCC	17043929
85.	CCCCCCCCCCC/C=C\CCCC(=O)O	75591877
86.	CCCCC=CCCCCCCCCCC(=O)O	17464796
87.	OCC(O)CO	155856911
88.	NC(=O)CC[C@H](N)C(=O)O	155850036
89.	CCCC/C=C/C(=O)O	8747438
90.	C=CC(O)C#CC#CCC=CCCCCCCC	16992263
91.	CCCCCC/C=C/CCCCCCCC(=O)O	8747474
92.	C=C(C)C(CC=C(C)C)COC(C)=O	17016827
93.	CCCCCCCCCCCCCCCCCCCCCCCCCCCC	17000772
94.	CCCCCCCCCCCCCCCCCC(=O)O	16994107

95.	CCCCCCCCCCCC=CCCCCCC(=O)O	4610418
96.	CCCCCC#CCC#CCCCCCCCC(=O)O	16990939
97.	CCCCCCCCCCCCCCCC(=O)O	16990058
98.	CCCCCCCCC=CCCCCCCCC(=O)O	16990039
99.	CCCCCC=CC=CC(=O)CCCCCCCC(=O)O	17160906
100.	CCCCC/C=C\C/C=C\CCCCCC(=O)O	52907274
101.	CCCC/C=C\CCCCCCCCCC(=O)O	8771765
102.	C=C[C@@H](O)C#CC#C[C@@H](O)/C=C\CCCCCCC	8746208
103.	CCCCCCCCCCCCCCC(=O)O	17002101
104.	CCCCCC=CCC=CCCCCCCCC(=O)O	16992839
105.	CCCCCCOC(=O)CCC	17005429
106.	CCC=CCC=CCC=CCCCCCC(=O)O	92108893
107.	CC/C=C\C/C=C\C/C=C\CCCCCC(=O)O	8771779
108.	CCCCCCCCCCCCCC(=O)O	16999435
109.	CCCCCCCCCCCCCCCCCCCC(O)CC(=O)O	8747650
110.	CCCCCCCCCCCCC(=O)O	17000880
111.	CC(O)C(=O)O	155823350
112.	CCCCC/C=C\C=C\CCCCCCCCC(=O)[O-]	44076887
113.	O=C(O)CCCCCCCC(=O)O	155861668
114.	CCCCCCCCCCCCCCCCC	17000762
115.	CCCCCCCCCCCCCCCC(O)CC(=O)O	8747636
116.	CCCCCCCCCCCCCCCCCCCCCCCCC(=O)O	16998942
117.	CCCCCCCCCC(=O)O	155804528
118.	CCCCCCOC(=O)CCCCC	17010312
119.	CCCCCCCCOC(=O)CCCCCCC	17044172
120.	CCCCCCCCCCCCCCCCCCCCCCCCCCO	17050399
121.	CCCCCCCCCCCC(=O)O	155804247
122.	CCCCCCCC/C=C/CCCCCCCC(=O)O	17547311
123.	Nc1nc2ncc(CNc3ccc(C(=O)N[C@@H](CCC(=O)O)C(=O)O)cc3)nc2c(=O)[nH]1	150658497
124.	CCCCCC=CCC=CCCCCCC(=O)O	4114417
125.	CCCCCCCCCCCCCCCCCCCCCCCCCC	17000771
126.	C=CC(O)C#CC#CC(O)C=CCCCCCCC	16992262
127.	CCCCCCCCCCCCCCCCCCCCCCCCCCCCCCO	17051120
128.	C=CC(=C)CCC=C(C)C	17017718
129.	CCCCCC/C=C\CCCCCCCCCCCC(=O)O	8771869
130.	CCCCC/C=C/C=C/C(=O)CCCCCCCC(=O)O	8747740
131.	O=CC(O)C(O)C(O)CO	155856930
132.	CCCCCCOC(=O)C(C)C	17004832

Table S4. Standardized SMILES and corresponding identification numbers (SynID) for cluster D

№	SMILES	SynID
1.	C=Cc1ccc(O)c(OC)c1	16989477
2.	OCc1ccccc1O	155854530
3.	Cc1ccc(C(C)C)c(O)c1	155788386
4.	OCCc1ccccc1	155855070
5.	CCCCCCC(C(=O)O)c1ccccc1	85081211
6.	O=C(O)CCCCCc1ccccc1	155861606
7.	CCCCCC(=O)OCCc1ccccc1	17044253
8.	O=C(O)c1ccc(O)cc1	155860456
9.	C=CCc1cc(OC)c(OC)c(OC)c1	16998739
10.	COc1cc(/C=C/C(=O)O)ccc1O	17385578
11.	CCCc1ccc(O)cc1	155800749
12.	CCCC(=O)OCCc1ccccc1	16996354
13.	COc1cc(C(=O)O)ccc1O	155839924
14.	Cc1ccc(C(=O)O)cc1	155790616
15.	COc1ccc(C(=O)O)cc1	155837699
16.	Cc1ccc(C(C)C)cc1	155788338
17.	CC(=O)Nc1ccc(S(=O)(=O)[O-])c2cc(S(=O)(=O)[O-])c(N=Nc3ccccc3)c(O)c12.[Na+].[Na+]	17145249
18.	Oc1ccccc1O	155851633
19.	COc1cc(C=O)ccc1O	155839463
20.	O=C(O)c1ccccc1	155859827
21.	O=C(O)Cc1ccccc1	155861226

Table S5. Standardized SMILES and corresponding identification numbers (SynID) for cluster E

№	SMILES	SynID
1.	Cc1cc2cc3c(C)cc(=O)oc3c(C)c2o1	16994397
2.	COc1c2ccoc2c(OCC=C(C)C)c2oc(=O)ccc12	17078318
3.	CC(C)(O)[C@H](O)COc1c2ccoc2cc2oc(=O)ccc12	17005439
4.	O=c1ccc2ccc3occc3c2o1	16999113
5.	COc1c(OC)c2occc2c2oc(=O)ccc12	16993683
6.	COc1c2ccoc2c(OC[C@@H](O)C(C)(C)O)c2oc(=O)ccc12	16998704
7.	O=c1ccc2cc3ccoc3cc2o1	16994953
8.	CC(C)=CCOc1c2occc2cc2ccc(=O)oc12	16998705
9.	CC(C)(O)[C@H](O)COc1c2occc2cc2ccc(=O)oc12	17054941
10.	COc1c2ccoc2cc2oc(=O)ccc12	16991340
11.	COc1c2ccoc2c(OC)c2oc(=O)ccc12	17050311
12.	COc1c2occc2cc2ccc(=O)oc12	16993011
13.	CC(C)=CCOc1c2ccoc2cc2oc(=O)ccc12	17050313
14.	COc1cc2ccc(=O)oc2cc1O	8745763
15.	O=c1ccc2ccc(O)cc2o1	155851421
16.	COc1ccc2ccc(=O)oc2c1CC=C(C)C	16998720
17.	CC1(C)OC1COc1c2ccoc2cc2oc(=O)ccc12	17132641
18.	COc1cc2ccc(=O)oc2c2ccoc12	17086900
19.	COc1ccc2nccc(C(=O)O)c2c1	17299952
20.	C=C(C)[C@@H](O)COc1c2ccoc2cc2oc(=O)ccc12	88198059
21.	CC(C)=CCC/C(C)=C/COc1c2ccoc2cc2oc(=O)ccc12	8867701
22.	COc1cc2occc2c2oc(=O)ccc12	17050314

Table S6. Main model quality parameters

	Parameter	Unit	Average	Metric*
Regression	Mouse Intravenous LD_{50}	Log10(mg/kg)	0.41 ± 0.01	RMSE
	Mouse Oral LD_{50}	Log10(mg/kg)	0.45 ± 0.01	RMSE
	Mouse Intraperitoneal LD_{50}	Log10(mg/kg)	0.49 ± 0.01	RMSE
	Mouse Subcutaneous LD_{50}	Log10(mg/kg)	0.65 ± 0.02	RMSE
	Mouse Skin LD_{50}	Log10(mg/kg)	0.81 ± 0.17	RMSE
	Mouse Intramuscular LD_{50}	Log10(mg/kg)	0.87 ± 0.09	RMSE
Classification	Carcinogenicity	-	0.79 ± 0.01	ROC AUC
	Hepatotoxicity	-	0.81 ± 0.17	ROC AUC
	Drug-induced liver injury (DILI)	-	0.90 ± 0.03	ROC AUC
	Cardiotoxicity	-	0.93 ± 0.01	ROC AUC

*These characteristics are taken from the Statistics section of the *Syntelly* platform (https://app.syntelly.com/statistics) for the period 01.07.2025. The data is presented as an average value ± SD (n = 5).

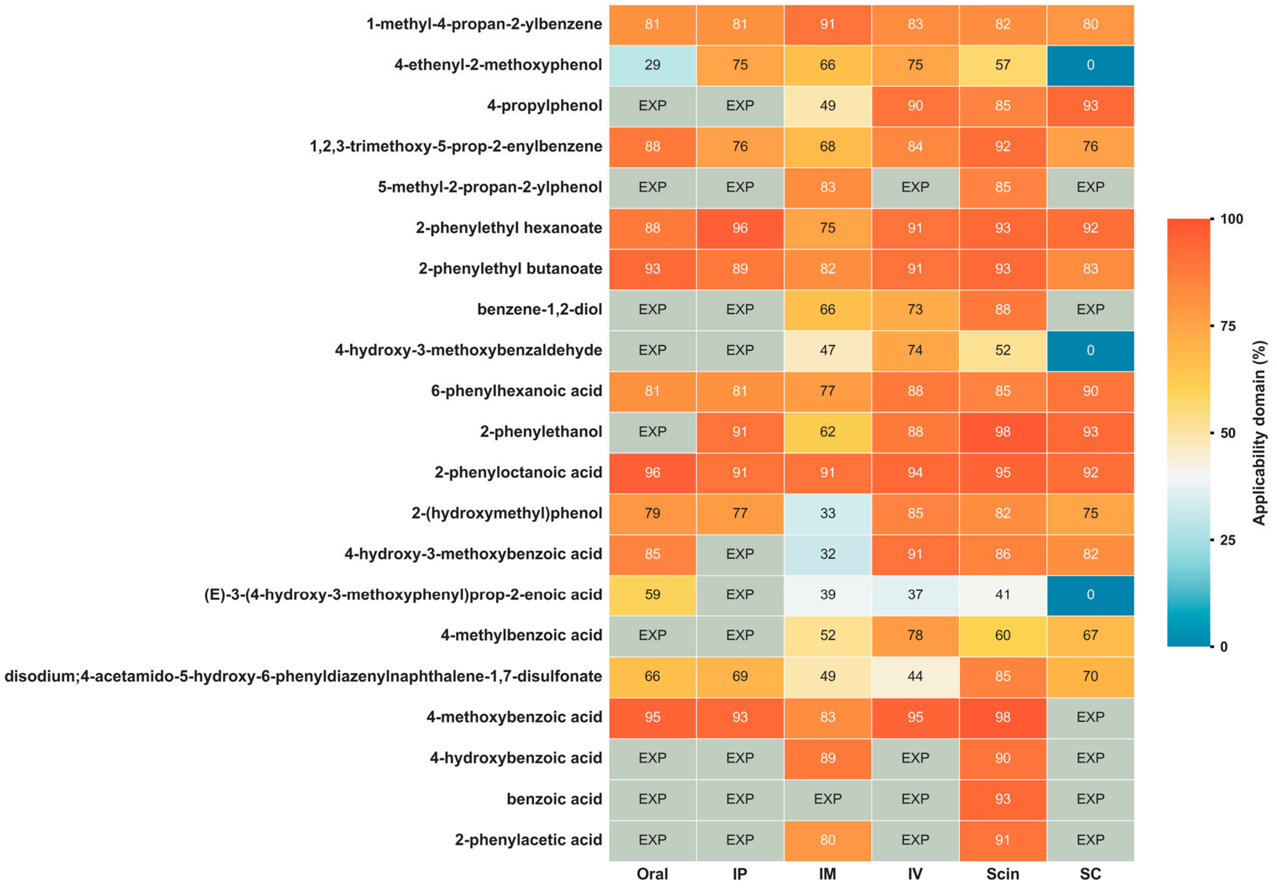

Figure S1. Heatmap of the values of the applicability domain (AD, %) for each predicted acute LD_{50} (mouse) of cluster D of *Heracleum sosnowskyi* with various routes of administration. EXP is the experimental values aggregated from the ChemIDplus, Toxic, PyTDC, ECHA, NIH, and PubMed platforms. The value 0 is the inability to predict the values for these molecules.

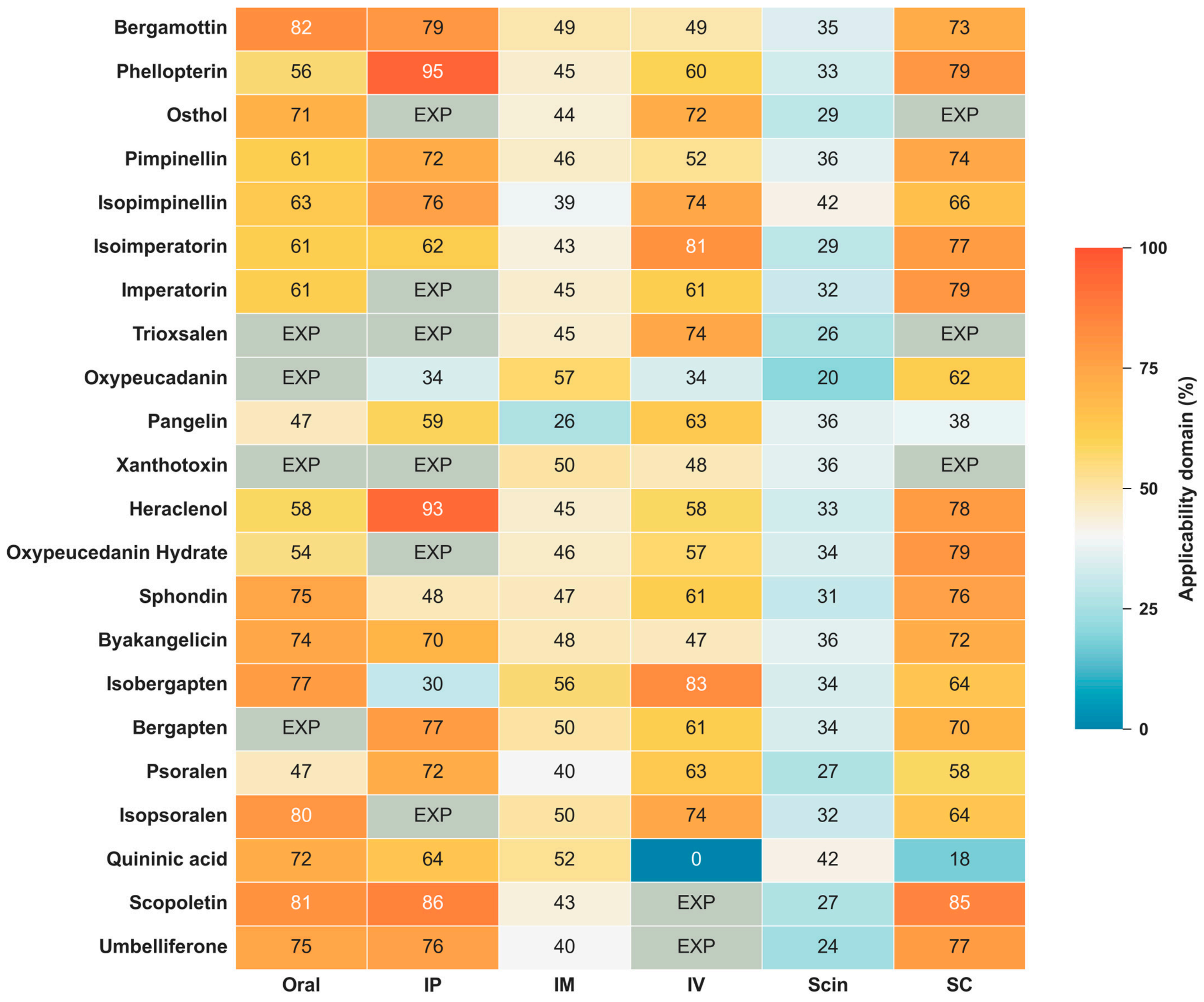

Figure S2. Heatmap of the values of the applicability domain (AD, %) for each predicted acute LD_{50} (mouse) of cluster E of *Heracleum sosnowskyi* with various routes of administration. EXP is the experimental values aggregated from the ChemIDplus, Toxic, PyTDC, ECHA, NIH, and PubMed platforms. The value 0 is the inability to predict the values for these molecules.
